# Comparison of four different hematocrit assays and the effect of albumin on their measurements

**DOI:** 10.3389/fvets.2022.937328

**Published:** 2022-08-24

**Authors:** Amelie Pare, Laura Kippen, Catherine Wagg, Matt Longmore, Soren Boysen

**Affiliations:** ^1^Department of Internal Medicine, Western Veterinary Specialist & Emergency Centre–VCA, Calgary, AB, Canada; ^2^Department of Emergency Care, Central Victoria Veterinary Hospital–VCA, Victoria, BC, Canada; ^3^Department of Veterinary Clinical and Diagnostic Sciences, Faculty of Veterinary Medicine, University of Calgary, Calgary, AB, Canada; ^4^Department of Emergency Care, Western Veterinary Specialist & Emergency Centre–VCA, Calgary, AB, Canada

**Keywords:** PCV, hematocrit, assays, anemia, conductivity, centrifugation, optical, impedance

## Abstract

Clinical decisions are influenced by hematocrit values. Centrifugation (reference standard), conductivity, optical and impedance methods are often used interchangeably to measure hematocrit. The effects of albumin, which are known to affect conductivity methods, have not been evaluated for limits of agreement (LOA) between hematocrit assays in small animals. Canine venous blood was collected from 74 clinical cases and measured by centrifugation (*n* = 72), conductivity (*n* = 73), impedance (*n* = 24) and optical (*n* = 50) methods. Bland-Altman analysis determined bias (± SD) and 95% LOA between methods. There was a statistically significant difference between centrifugation hematocrit values and values obtained *via* conductivity (*p* < 0.0001), optical (*p* < 0.0001), and impedance (*p* = 0.0082) methods. The conductivity method underestimated hematocrit by 2.1 ± 2.9% (95% LOA −3.54 to 7.88), the optical method by 3.1 ± 3.6% (95% LOA −4.0 to 10.2), and the impedance method by 2.3 ± 3.7% (95% LOA −5 to 9.6) when compared to centrifuged hematocrit values. The hematocrit difference between conductivity and centrifugation methods was statistically different for low (4%, 0–5%), within reference limits (3%, −5 to 8%), and high (2%, −2 to 5%) albumin values, respectively (*p* = 0.02), with *post-hoc* analysis demonstrating that the difference occurred between the low and high albumin groups. This study confirms that albumin values outside reference limits can affect the conductivity method and that hematocrit values obtained *via* conductivity, optical and impedance methods underestimate values obtained *via* centrifugation. Therefore, the hematocrit methods cannot be used interchangeably. The wide limits of agreement also demonstrates that care must be taken when making clinical decisions with different hematocrit methodologies.

## Introduction

Veterinary patients with erythrocytosis or anemia often require serial measurement of their blood hematocrit (Hct) to optimize diagnostic and treatment options. Veterinary hospitals often use hematology devices interchangeably, even if the devices use different methodologies. This creates a need to determine if Hct assays produce the same value in a variety of clinical settings, as an inaccurate result can affect clinical decisions and patient outcomes.

There are four common assays used to measure blood hematocrit values: centrifugation, conductivity, optical reflectance and impedance (1). Centrifugation is considered the reference standard: often referred to as the microhematocrit or packed cell volume (PCV), it separates the cellular components of blood, allowing measurement of packed erythrocytes. It can be affected by excessive collection tube anticoagulant and improper sample preparation such as a prolonged delay between sampling and measurement, or extended storage times at room temperature (2). Conductivity measures the difference between the plasma, which transmits electricity, and the erythrocytes, which are non-conducting cells (1). Therefore, when factors that affect conductivity (sodium, chloride, albumin, white blood cells, lipemia, anticoagulant and intravenous fluid therapy) become abnormal, the Hct measured may be inaccurate (3–8). Optical measurement uses light transmittance and the absorbance spectra of red blood cells to determine the hematocrit (1). Impedance measures the number of changes in electrical resistance passing through a specific orifice to determine the number of red blood cells (9). Both optical and impedance assays can be affected by abnormal cell size and autoagglutination (10).

Although evaluated in human medicine, few studies in veterinary medicine have assessed the limits of agreement (LOA) between different Hct assays. A recent study showed that there are wide LOA between Hct values obtained from dogs and cats *via* optical point-of-care (POC) measurement and complete blood count (CBC) values, which means that the bias between the two methods is clinically relevant and that comparison of the results is unreliable (11). In the same study, lipemia, icterus, autoagglutination, hemolysis and reticulocyte count did not affect Hct values, however, to the authors' knowledge, the influence of albumin on Hct measurement in veterinary medicine is unknown. The objectives of the current canine study were to determine the LOA between centrifugated, conductivity, optical and impedance blood Hct values and to assess the effect of albumin on Hct measurement for the different methodologies. We hypothesized that albumin values outside reference limits would cause inaccurate values for the conductivity-based methods in dogs.

## Methods

### Study population and case selection

Owner consent was obtained for all patients enrolled in the study. This research project was approved by the University of Calgary Animal Care Committee (File # AC12-0027) and funding was provided by the University curriculum office. Dogs presenting to one of two private emergency and referral veterinary hospitals in Calgary, Alberta, Canada that required measurement of Hct and albumin as part of their initial evaluation, were enrolled.

### Sampling and blood analysis

Direct venous blood samples were obtained by registered animal health technicians using either cephalic, lateral saphenous, or jugular venipuncture. Needle and syringe size were selected at the discretion of the attending technician. All dogs required blood sampling of varying volumes (depending on the reason for blood draw) as part of their routine work up, and no additional blood was collected for the purpose of this study. Whole blood was divided and stored in 1 or 3 ml ethylenediaminetetraacetic acid (EDTA) blood collection tubes for CBC analysis and/or 3 ml serum-separating tube for biochemistry analysis; samples were refrigerated immediately after sampling and prior to analysis when required. Blood samples placed in serum separator tubes were allowed to coagulate, then centrifuged at 1,500 revolutions per minute (rpm) for 5 min. Upon completion of centrifugation the serum was transferred to a sterile red top tube using a plastic transfer pipette. For conductivity samples, whole blood was transferred from the collection syringe to either a 1 ml lithium heparin tube or heparinized microhematocrit tube and immediately analyzed. Albumin was measured using serum *via* the bromcresol green dye-binding method. EDTA samples were used for the impedance, optical and centrifugation Hct measurements. Dogs had Hct values measured using an in-house centrifugation device (Unico, model C-MH30; Dayton, New Jersey) and conductivity values measured using an in-house handheld device (I-Stat 1 analyzer, model MN300, Abaxis Veterinary Diagnostics; Union City, California). The conductivity device was calibrated automatically as part of each test cycle, using control solutions provided with each lot of cartridges. The optical (ADVIA 120, Siemens Healthcare Diagnostics, Deerfield, Illinois) and impedance (HemaTrue analyzer, Heska; Loveland, Colorado) measurements were done using an offsite laboratory analyzer. The blood and serum were refrigerated during transportation and were analyzed within 24 h of sampling.

### Statistical analysis

**Figure 1:**
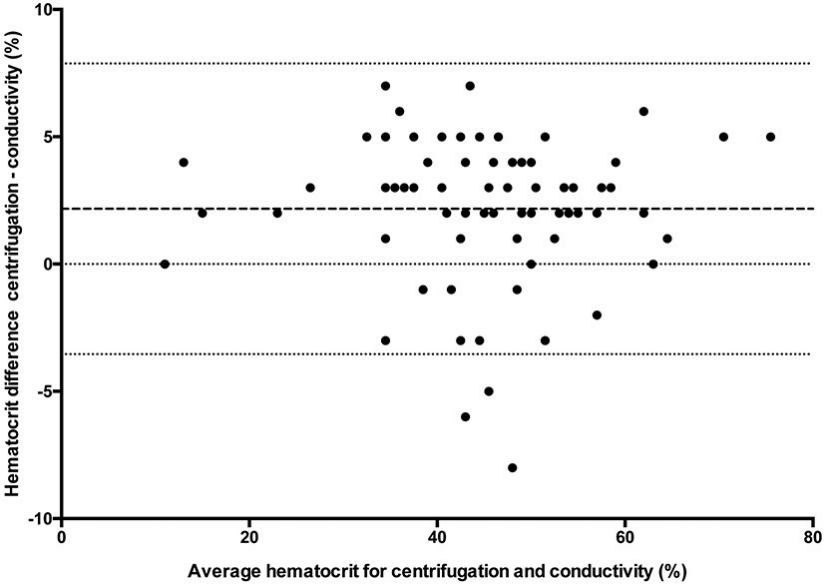
Comparison of mean bias (bolder dotted line) and 95% limit of agreement (outer dotted lines) between the centrifuged hematocrit value and conductivity method.

**Figure 2:**
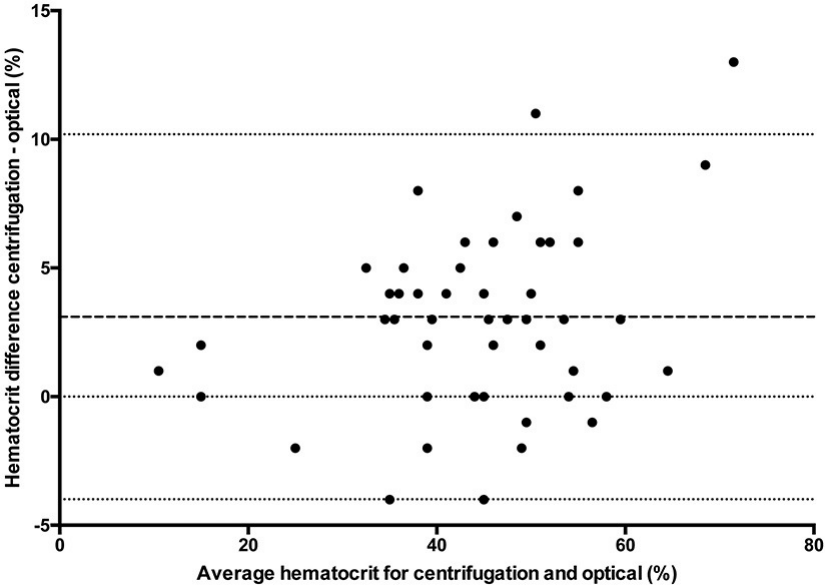
Comparison of mean bias (bolder dotted line) and 95% limit of agreement (outer dotted lines) between the centrifuged hematocrit value and optical method.

**Figure 3:**
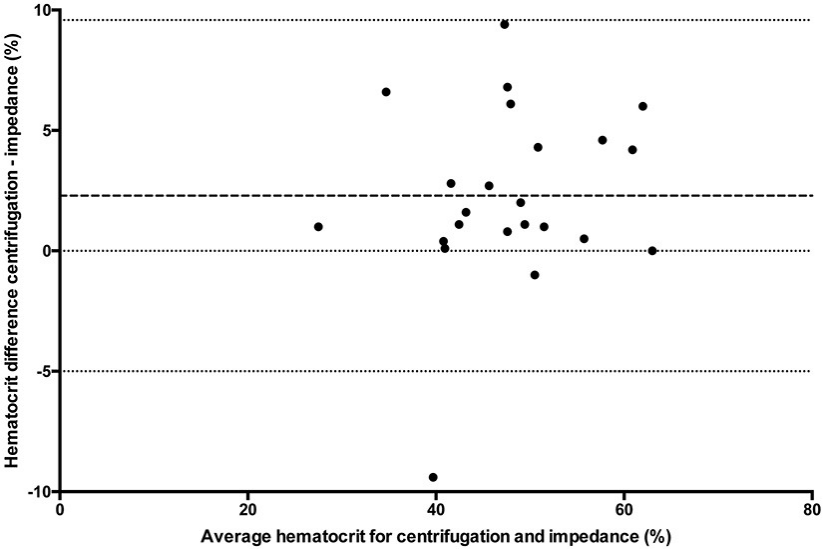
Comparison of mean bias (bolder dotted line) and 95% limit of agreement (dotted lines) between the centrifuged hematocrit value and impedance method.

All statistical analyses were performed using Prism software (GraphPad Prism version 5.0; San Diego, California). Data was tested for normality using the Kolmogorov-Smirnov test and reported as mean ± standard deviation (SD) when normally distributed, and median and range when not normally distributed. One way ANOVA (passed normalcy) or Kruskal-Wallis (failed to pass normalcy) with *post-hoc* Tukey's tests were used for multiple comparisons when more than two analyses were performed on each blood sample. A *P* ≤ 0.05 was considered statistically significant. Hct values for the conductivity, optical, and impedance devices were compared to centrifugation values using paired *t*-tests (passed normalcy) or Wilcoxon signed rank (failed to pass normalcy) and Bland-Altman plots to generate a mean bias ± SD and 95% LOA (Figures 1–3). Centrifugation was considered the reference standard for Hct comparisons, as it is often used as quality control to calibrate other methods (9). To determine the effect of albumin, samples were grouped based on the following albumin values; low if <30 g/L, within reference limits if 30–40 g/L, and elevated if > 40 g/L. Pearson's correlation coefficient was used to test for correlations between Hct values and albumin concentration.

## Results

Seventy-four dogs met the inclusion criteria over the 25-month study period. Centrifugation Hct values were recorded in 72/74 dogs, conductivity in 73/74, optical in 50/74 and impedance in 25/74. One impedance value was excluded as an outlier as it was double the Hct value derived from the other two concurrent analytical methods, leaving 24 impedance results for analysis. Of the 74 dogs, 71 had albumin concentration available for analysis, 41 of which were within reference limits, 20 were decreased, and 10 had elevated values. All data passed normalcy with the exception of conductivity methods when albumin levels were within reference limits. There were 48 samples that had paired conductivity, optical and centrifuged Hct evaluations and 23 samples that had paired conductivity, impedance and centrifugation Hct evaluations. There was only one blood sample that had all four analyses performed. The number of direct paired sample comparisons to centrifugation are included in Table 1.

**Table 1:** Hct values and Bland-Altman difference with 95% LOA for three methodologies and for cases with low, within reference limit, and high albumin levels compared to centrifugation.

	**Centrifugation**	**Conductivity**	**Optical**	**Impedance**
Mean Hct (%)	46.8 (*n* = 73)	44.3 (*n* = 72)	42.8 (*n* = 50)	46.2 (*n* = 24)
±SD (%)	11.9	11.7	11.7	8.5
*P*-value if compared with centrifugation methodology	N/A	***P*** **< 0.0001 (*****n*** **= 71)**	***p*** **< 0.0001 (*****n*** **= 49)**	***p*** **= 0.0073 (*****n*** **= 23)**
95% CI of the mean difference	N/A	1.5–2.9	2.1–4.1	0.7–3.9
Bland-Altman difference in Hct compared to centrifugation value ± SD (%)	N/A	2.2–2.9 (*n* = 71)	3.1 ± 3.6 (*n* = 49)	2.3–3.7 (*n* = 24)
95% LOA	N/A	−3.5 to 7.9	−4.0 to 10.1	−5 to 9.6
Low albumin				
Mean Hct (%)	43 (*n* = 19)	39.4 (*n* = 20)	39.3 (*n* = 16)	42.9 (*n* = 4)
±SD (%)	15.4	14.5	14	11.8
*P*-value if compared with centrifugation methodology	N/A	***P*** **< 0.0001 (*****n*** **= 19)**	***P*** **= 0.0028 (*****n*** **= 15)**	***P*** **= 0.0479 (*****n*** **= 4)**
95% CI of the mean difference	N/A	3.0–4.3	1.3–5.0	0.6–7.2
Bland-Altman difference in Hct compared to centrifugation value ± SD (%)	N/A	3.7 ± 1.3 (*n* = 19)	3.1 ± 3.4 (*n* = 15)	3.6 ± 2.2 (*n* = 4)
95% LOA	N/A	to 6.3	−3.5 to 9.7	0.8. to 8.0
Albumin within reference limits				
Hct (%)	Mean 48.4 (*n* = 40)	Median 48 (*n* = 40)	Mean 43.7 (*n* = 29)	Mean 48.7 (*n* = 12)
Variation (%)	±10.4 SD	Range 14–64	±10.2 SD	±9.5 SD
*P*-value if compared with centrifugation methodology	N/A	***P*** **=** **0.0008 (*****n*** **=** **39)**	***P*** **=** **0.0002 (*****n*** **=** **29)**	***P*** **=** **0.007 (*****n*** **=** **11)**
95% CI of the mean difference	N/A	N/A	1.6–4.5	1.1–5.2
Bland-Altman difference in Hct compared to centrifugation value ±SD (%)	N/A	1.9 ± 3.3 (n = 39)	3.0 ± 3.8 (n = 29)	3.1 ± 3.1 (*n* = 11)
95% LOA	N/A	−4.6 to 8.4	4.4 to 10.5	−2.9 to 9.1
High albumin				
Mean Hct (%)	49.6 (*n* = 10)	47.9 (*n* = 10)	49.2 (*n* = 5)	46.1 (*n* = 5)
±SD (%)	10.4	10.1	10.9	3.6
*P*-value if compared with centrifugation methodology	N/A	***P*** **=** **0.02 (*****n*** **=** **10)**	*P* = 0.14 (*n* = 5)	*P* = 0.88 (*n* = 5)
95% CI of the mean difference	N/A	0.4 to 3.1	−1.8 to 8.6	−7.3 to 8.2
Difference in Hct compared to centrifugation value ±SD (%)	N/A	1.7% ± 1.9 (*n* = 10)	0.5% ± 6.2 (*n* = 5)	3.4% ± 4.2 (*n* = 5)
95% LOA	N/A	−2.0 to −5.4	−11.8 to 12.7	−4.8 to −11.6

*Hct, hematocrit; SD, standard deviation; LOA, limits of agreement. Bold characters indicate a statistically significant difference*.

For multiple comparisons, there was a statistically significant difference (*p* < 0.0001) between conductivity, optical and centrifuged samples (*n* = 48) with *post-hoc* analysis demonstrating a difference between centrifugation and conductivity and between centrifugation and optical methods. There was no statistical difference between conductivity and optical methods. There was a statistically significant difference (*n* = 23) between conductivity, impedance, and centrifugation samples (*p* = 0.0082) with *post-hoc* analyses demonstrating the difference existed between conductivity and centrifugation and between impedance and centrifugation. There was no significant difference between conductivity and impedance methods. Hematocrit values and direct comparison between centrifugation and each of the other methodologies are reported in Table 1. There were insufficient samples to compare impedance and optical methods (*n* = 1).

### Hypoalbuminemia

Within the low albumin group there were 15 samples that had paired conductivity, optical and centrifugation Hct evaluations, and four samples that had paired conductivity, impedance and centrifugation Hct evaluations. There was a statistically significant difference between the conductivity, optical and centrifugation methods (*P* < 0.0004), with *post-hoc* analysis demonstrating the difference between the centrifugation and conductivity method and the centrifugation and optical method. The multiple comparison test did not detect any statistical differences between the conductivity, impedance and centrifugation Hct evaluations (*p* = 0.08). There was no statistical difference between conductivity and optical methods, or between conductivity and impedance methods. There were no hypoalbuminemic samples that analyzed both optical and impedance methods. Hematocrit values and comparison between centrifugation and each of the other methodologies for cases with low albumin levels are reported in Table 1.

### Albumin value within reference limits

In dogs that had albumin concentrations within reference limits there were 28 samples with paired conductivity, optical and centrifugation Hct evaluations, and 11 samples with paired conductivity, impedance and centrifugation Hct evaluations. The multiple comparisons test showed a statistically significant difference between conductivity, optical and centrifugation (*p* = 0.0002) and between conductivity, impedance and centrifugation Hct evaluations (*P* = 0.006) with *post-hoc* analysis showing the differences were between centrifugation and all 3 other methodologies. There was no statistical difference between conductivity and optical methods, or between conductivity and impedance methods. Only one case with albumin concentration within reference limits had all 4 analytical methods used. Hematocrit values and comparison between centrifugation and each of the other methodologies for cases with albumin values within reference limits are reported in Table 1.

### Elevated albumin

Within the high albumin group there were 5 samples that had paired conductivity, optical and centrifugation Hct evaluations, and 5 samples that had conductivity, impedance and centrifugation Hct evaluations. There were no statistically significant differences in any of the multiple comparisons between groups. Hematocrit values and comparison between centrifugation and each of the other methodologies for cases with high albumin levels are reported in Table 1.

### Degree of Hct bias and albumin concentration

A multiple comparisons test for the 3 different albumin groups demonstrated a statistically significant difference (*p* = 0.02) in the degree of Hct bias between conductivity and centrifugation (Table 2). For conductivity methods, there was no statistically significant correlation between the Hct bias and albumin values (*r* = 0.112). The degree of Hct bias between centrifuge and both optical and impedance methods was not statistically significant for different albumin concentrations, nor were the correlations between Hct and albumin concentrations for these methodologies.

**Table 2:** Effect of albumin on the hematocrit bias for conductivity compared to centrifuge methods.

**Albumin value**	**Median difference in Hct between conductivity and centrifugation % (LOA)**
Low	4 (0–5)
Within reference limits	3 (−5 to 8)
High	2 (−2 to −5)
***P*** -value^*^	**0.02**

* *post-hoc analysis demonstrated that the difference occurred between the low and high albumin groups. Bold characters indicate a statistically significant difference*.

## Discussion

The results of this study show that there are wide LOA between centrifugation, conductivity, optical and impedance blood Hct values. The hematocrit values were significantly underestimated by all other assays when compared to the centrifugation method. The study also demonstrated that albumin concentration affects the degree of bias for conductivity-based methodologies.

The large standard deviations from each assay and wide LOA in each comparison group indicate that there are large discrepancies on a sample-by-sample evaluation. Reported SD in humans varies between 4.7 and 5.9% for centrifugation, 6.2–8.7% for conductivity (4, 6) and is reported at 7.4% for optical (12) and 4.5% for impedance methods (4). As demonstrated in Table 1, SD in the current study was higher than previously reported. However, this was not an unexpected finding as it is consistent with a recent study that showed large SD for a laboratory-based analyzer (±15.7%) (11). Wide LOA are problematic because discrepancies of >5%, as seen in the current study, can affect clinical decisions regarding treatment options. For example, the most recent American College of Veterinary Internal Medicine consensus statement on immune mediated hemolytic anemia recommends a second line of immunosuppressive therapy when there is a decrease in the hematocrit of ≥5% within 24 h (13). It was stated that a 5% difference should be sufficient to account for measurement errors, although results of the current study suggest this may not be the case. As such, clinicians should use the same assay when performing serial Hct measurements, consider possible differences between different assays, and integrate any results with the patient's clinical status when making treatment decisions.

Our findings also suggest that different methodologies underestimated Hct values by 2.17, 3.1, and 2.3% (conductivity, optical and impedance, respectively) when compared to centrifugation and that this difference is statistically significant. These findings are consistent with previous studies that demonstrated Hct values from dogs and cats measured *via* impedance differ significantly from centrifuge values (14, 15). Variable Hct values may lead to unnecessary treatments including blood transfusions, which can carry serious risks to patients and additional costs to owners (16, 17). Another study showed Hct values obtained from an automated method could not be substituted for a centrifugation-based method (14). These findings confirm that serial monitoring of Hct value should be conducted with the same assay and that the decision to transfuse, or change treatments, should ideally be based on centrifuge methods and individual patient evaluation.

Lastly, it was hypothesized that albumin levels would influence the conductivity method. Although the bias in Hct compared to centrifugation tended to persist at different albumin levels for all methodologies, the bias became larger at lower albumin concentrations and smaller at higher albumin concentrations for the conductivity method. In contrast, the Hct bias between centrifuge and both optical and impedance methods was not statistically significant for different albumin concentrations. This finding is similar to what is reported in studies on human cardiopulmonary bypass patients who undergo massive hemodilution, which show that hypoalbuminemia exacerbates the inaccuracy of the conductivity method (4, 6, 18). For every 1 g/dL decrease in total protein there is a 1% decrease in the conductivity Hct reading, which is reversed when patients are treated with human albumin (18). This suggests that as protein decreases conductivity Hct accuracy worsens with the bias becoming greater (18). In veterinary patients, this underestimation is also reported when comparing conductivity to centrifuge methods (15). The greater Hct bias at lower albumin concentrations is similar to what was found in the current study, which also demonstrated that as albumin levels increase the expected Hct bias compared to centrifugation also decreases. The optical and impedance methods are not reported to be affected by hypoalbuminemia or other factors that affect conductivity (1, 9, 19). Since the morphological appearance of the red blood cells, the presence of agglutination and the sodium value were not considered, it is possible that those also contributed to this difference, or that those markers are more significant in the face of different albumin concentrations. Therefore, the true significance of this finding is undetermined. Furthermore, given the small sample size of hyperalbuminemic patients in the impedance and optical groups, it is possible that a difference in the degree of bias also exists with different albumin levels, but was not detected due to a type II error. However, the results suggest that conductivity based methods should be interpreted with caution in patients with abnormal albumin concentration, and other methodologies not influenced by albumin concentration should be considered, with the limitations stated previously.

There are several limitations in the current study. All dogs did not have Hct values assessed by all four methodologies. Albumin concentration was also not measured in all patients. Available samples were collected for diagnostic purposes at the discretion of the attending clinician and the quantity of blood collected was sometimes limited by patient size. These factors limited the number of cases within each subcategory and a larger number of cases may have changed the variability and statistical differences between groups. Abnormal sodium values can also affect both conductivity based and optical based methods (5, 20), but the number of cases with abnormal sodium values in the current study was insufficient to allow for statistical analysis between the different methodologies. Also, lipemia, icterus, autoagglutination and hemolysis were not considered, which may also have contributed to the wide LOA noted between groups.

## Conclusion

Impedance, optical and conductivity methods of Hct measurement are statistically different from the reference standard of centrifugation and should not be used interchangeably, as such differences may be clinically relevant. In addition, conductivity-based methods are affected by abnormal albumin concentration and the choice of Hct assay should be chosen considering possible patient factors that can influence different methodologies.

## Data availability statement

The original contributions presented in the study are included in the article/Supplementary material, further inquiries can be directed to the corresponding author.

## Ethics statement

The animal study was reviewed and approved by University of Calgary Animal Care Committee (File # AC12-0027). Written informed consent was obtained from the owners for the participation of their animals in this study.

## Author contributions

SB, CW, ML, and LK contributed to the conception and design of the study. SB organized the database and performed the statistical analysis. LK and ML collected data and wrote the first draft of the manuscript. AP assisted with statistical analysis and wrote the final sections of the manuscript. All authors contributed to writing manuscript revisions and approved the submitted version of the article.

## Funding

This study was funded by the University curriculum office.

## Conflict of interest

The authors declare that the research was conducted in the absence of any commercial or financial relationships that could be construed as a potential conflict of interest.

## Publisher's note

All claims expressed in this article are solely those of the authors and do not necessarily represent those of their affiliated organizations, or those of the publisher, the editors and the reviewers. Any product that may be evaluated in this article, or claim that may be made by its manufacturer, is not guaranteed or endorsed by the publisher.
